# Bone Marrow-Derived Mesenchymal Stromal Cell Therapy in Severe COVID-19: Preliminary Results of a Phase I/II Clinical Trial

**DOI:** 10.3389/fimmu.2022.932360

**Published:** 2022-07-04

**Authors:** Céline Grégoire, Nathalie Layios, Bernard Lambermont, Chantal Lechanteur, Alexandra Briquet, Virginie Bettonville, Etienne Baudoux, Marie Thys, Nadia Dardenne, Benoît Misset, Yves Beguin

**Affiliations:** ^1^ Department of Clinical Hematology, University Hospital Center of Liège, Liège, Belgium; ^2^ Hematology Research Unit, Groupe Interdisciplinaire de Génoprotéomique Appliquée - Infection, Immunité & Inflammation (GIGA-I3), Groupe Interdisciplinaire de Génoprotéomique Appliquée (GIGA) Institute, University of Liège, Liège, Belgium; ^3^ Department of Intensive Care, University Hospital Center of Liège, Liège, Belgium; ^4^ Laboratory of Cardiology, Groupe Interdisciplinaire de Génoprotéomique Appliquée (GIGA) Institute, University of Liège, Liège, Belgium; ^5^ Groupe Interdisciplinaire de Génoprotéomique Appliquée (GIGA)-In silico Medicine, University of Liège, Liège, Belgium; ^6^ Laboratory of Cell and Gene Therapy, University Hospital Center of Liège and University of Liège, Liège, Belgium; ^7^ Department of Medico-Economic Information, University Hospital Center of Liège, Liège, Belgium; ^8^ University Hospital Center of Biostatistics, Faculty of Medicine, University of Liège, Liège, Belgium

**Keywords:** mesenchymal stromal cells, cellular therapy, COVID-19, SARS-CoV-2, acute respiratory distress syndrome, intensive care unit (ICU)

## Abstract

**Background:**

Treatment of acute respiratory distress syndrome (ARDS) associated with COronaVIrus Disease-2019 (COVID-19) currently relies on dexamethasone and supportive mechanical ventilation, and remains associated with high mortality. Given their ability to limit inflammation, induce immune cells into a regulatory phenotype and stimulate tissue repair, mesenchymal stromal cells (MSCs) represent a promising therapy for severe and critical COVID-19 disease, which is associated with an uncontrolled immune-mediated inflammatory response.

**Methods:**

In this phase I-II trial, we aimed to evaluate the safety and efficacy of 3 intravenous infusions of bone marrow (BM)-derived MSCs at 3-day intervals in patients with severe COVID-19. All patients also received dexamethasone and standard supportive therapy. Between June 2020 and September 2021, 8 intensive care unit patients requiring supplemental oxygen (high-flow nasal oxygen in 7 patients, invasive mechanical ventilation in 1 patient) were treated with BM-MSCs. We retrospectively compared the outcomes of these MSC-treated patients with those of 24 matched control patients. Groups were compared by paired statistical tests.

**Results:**

MSC infusions were well tolerated, and no adverse effect related to MSC infusions were reported (one patient had an ischemic stroke related to aortic endocarditis). Overall, 3 patients required invasive mechanical ventilation, including one who required extracorporeal membrane oxygenation, but all patients ultimately had a favorable outcome. Survival was significantly higher in the MSC group, both at 28 and 60 days (100% vs 79.2%, p = 0.025 and 100% vs 70.8%, p = 0.0082, respectively), while no significant difference was observed in the need for mechanical ventilation nor in the number of invasive ventilation-free days, high flow nasal oxygenation-free days, oxygen support-free days and ICU-free days. MSC-treated patients also had a significantly lower day-7 D-dimer value compared to control patients (median 821.0 µg/L [IQR 362.0-1305.0] vs 3553 µg/L [IQR 1155.0-6433.5], p = 0.0085).

**Conclusions:**

BM-MSC therapy is safe and shows very promising efficacy in severe COVID-19, with a higher survival in our MSC cohort compared to matched control patients. These observations need to be confirmed in a randomized controlled trial designed to demonstrate the efficacy of BM-MSCs in COVID-19 ARDS.

**Clinical Trial Registration:**

(www.ClinicalTrials.gov), identifier NCT04445454

## Introduction

Severe forms of COronaVIrus Disease-2019 (COVID-19) caused by Severe Acute Respiratory Syndrome CoronaVirus-2 (SARS-CoV-2) are associated with a mortality of up to 30% in critically ill patients hospitalized in the intensive care unit (ICU) (1). SARS-CoV-2, through its spike (S) protein, uses the angiotensin-converting enzyme 2 (ACE2) and the transmembrane protease serine 2 (TMPRSS2) to infect human cells (2). Entry of the virus and its replication within infected cells cause both epithelial and endothelial cell damages, that can result in alveolitis with pulmonary oedema and endothelial inflammation with pulmonary intravascular coagulopathy, therefore affecting both lung ventilation and perfusion (3). Release of inflammatory molecules by damaged cells and alveolar macrophages is responsible for the recruitment of neutrophils, activated monocytes and T cells (3). In most patients, disease is mild to moderate, and the initial inflammatory response is rapidly followed by a highly efficient adaptative immune response, with plasmablast proliferation and production of neutralizing antibodies, but also robust CD4^+^ and CD8^+^ T-cell responses (4–7). However, some patients experience a severe disease associated with high viral load (8) and inappropriate immune responses (T-cell lymphopenia and exhaustion, skewing of lung immune responses towards proinflammatory CD8^+^ and Th17 cells and monocyte-derived proinflammatory macrophages) (5, 8–10), high amounts of cytokines and chemokines including interferon gamma-inducible protein (IP)-10, interleukin (IL)-6 and IL-10 (7), and intravascular coagulopathy indicated by high levels of D-dimers (11).

Several drugs (such as remdesivir, hydroxychloroquine, lopinavir, interferon) have failed to improve the outcome of these critically ill patients (12, 13). Neutralizing monoclonal antibodies are mostly effective in patients with non-severe disease at high risk of evolution toward a severe/critical disease, and their efficacy depends on the SARS-CoV-2 variant. To date, the only drugs that have reduced mortality in COVID-19 ARDS are anti-inflammatory/immunosuppressive drugs: dexamethasone (14), IL6 antagonists (tocilizumab or sarilumab) (15) and the JAK inhibitor baricitinib (16). In routine clinical practice, better understanding of the disease, allowing optimization of oxygen support for COVID-19 patients, and the generalized use of dexamethasone in severe COVID-19 has allowed a reduction in mortality between the first and second waves, but the ICU mortality rate remains high (17).

The interest of mesenchymal stromal cell (MSC) therapy in COVID-19 ARDS arises from their ability to both mitigate immune responses (18) and promote tissue regeneration (19). These non-hematopoietic multipotent progenitors can be easily isolated from several human tissues and have demonstrated their safety in many clinical trials in several diseases, including graft-versus-host-disease and Crohn’s disease (20, 21). MSCs modulate both adaptive and innate immune cells, the main effects being on T cells and macrophages. Their potency is enhanced in inflammatory environments (mostly in the presence of IFN-γ, TNF-α, IL-1α and/or IL-1β) (22), while their migration capacities and their ability to secrete trophic factors (such as VEGF) are increased when exposed to other environmental factors such as hypoxia (23, 24).

These properties, associated with first reports of the efficacy of MSC infusions in COVID-19 pneumonia (25, 26), prompted us to initiate a phase I/II clinical trial evaluating the safety and efficacy of bone marrow (BM)-derived MSCs for severe COVID-19 infection. In this preliminary report of our study, we describe the outcome of 8 patients treated with BM-MSCs, and retrospectively compare them with matched control patients.

## Materials and Methods

### Patient Selection

Patients were eligible if they met the following criteria: age between 18 and 70 years, microbiologically or radiologically confirmed COVID-19 pneumonia (as defined by an extensive interstitial pneumonia consistent with viral pneumonia on CT scan within 10 days prior to randomization, and positive result of COVID-19 polymerase chain reaction (PCR) test within 14 days prior to inclusion), and requiring oxygen administration (SpO2 ≤ 93% on room air) in the ward or intensive care unit (ICU). In this first part of the study, only ICU patients were included. Exclusion criteria were: ongoing pregnancy, extracorporeal membrane oxygenation (ECMO), limitations to intensity of care, life expectancy inferior to 24 hours, known allergy to components of the investigational medicinal product (IMP), pre-existing bone marrow transplantation or immunosuppressive therapy, active secondary infection, any malignancy (except non-melanoma skin carcinoma) within 2 years before inclusion, pre-existing thrombo-embolic pathology, and participation in another clinical trial (use of anti-viral/supportive drugs for COVID-19 infection on a compassionate use basis was not an exclusion criterion).

The study protocol conformed to the ethical guidelines of the 1975 Declaration of Helsinki and was approved by the local Ethics Committee of the University Hospital of Liège, the centralized Ethics Committee of the Cliniques Universitaires Saint-Luc, and the Belgian Federal Agency for Medicines and Health Product (EudraCT: 2020-002102-58; ClinicalTrials.gov identifier: NCT04445454). Signed informed consent was obtained from participating subjects, or – if impossible (clinical condition precluding capacity to consent) - from his/her legal representative.

### Mesenchymal Stromal Cells

MSC donors were healthy adult volunteers, unrelated to the recipient. No HLA matching between patient and donor was required. Bone marrow collection and MSC expansion cultures were carried out at the Laboratory of Cell and Gene Therapy (LTCG) at the University of Liège according to good manufacturing practice (GMP) standards, as previously described (27, 28). Detailed procedures for culture and quality control are provided in **Supplementary Material**. Briefly, after bone marrow collection, mononuclear cells were isolated and the cell suspension was seeded at a density of 160,000 cells/cm². On day 14 and 21, MSCs were trypsinized and replated at a lower density (4,000 cells/cm^2^), and cultured until passage 3, then cryopreserved. Population doubling level (calculated between passage 1 and 3) ranged from 3.6 to 6.2 (median 5.1). Quality controls included morphology, identity and purity (phenotype by flow cytometry), karyotype, viability, and immunosuppressive properties, as well as sterility tests (**Supplementary Table 1** in **Supplementary Material**). MSCs wererapidly thawed in a water bath at 37°C and diluted in the Laboratory of Cell and Gene Therapy (LTCG) and infused within 1 hour of thawing.

### Design of the Study and Follow-Up

Eligible subjects were scheduled to receive 3 infusions of 1.5-3x10^6^ BM-MSCs/kg, at 3 (± 1) day intervals. Patients received anticoagulant therapy, cetirizine and paracetamol before MSC infusion. Potential toxicities associated with MSC infusions were carefully monitored and recorded on the appropriate infusion form. Follow-up included daily assessment of vital status and vital signs until discharge as per institutional protocol.

The following clinical data were collected within 24 hours of ICU admission: age, body mass index (BMI), PaO_2_/FiO_2_ ratio, Sepsis-related Organ Failure Assessment (SOFA) score, viral load, and several biological values (including lymphocyte count, C-reactive protein (CRP), ferritin, D-dimers, creatinine). Outcomes were safety, death at day 28 and at day 60, ICU-free days (number of days out of ICU within 28 days from admission to ICU and after last discharge from ICU), need for mechanical ventilation (MV), number of invasive ventilation-free days (number of days without invasive ventilation after the last extubation within 28 days of invasive ventilation initiation), high-flow nasal oxygen (HFNO)-free days (number of days without HFNO after the last weaning within 28 days of HFNO initiation), and O_2_ support-free days (number of days without any oxygen support after the last oxygen administration within 28 days of oxygen support initiation), need for noradrenaline, day-7 CRP and D-dimers values.

Using our database of COVID-19 patients treated at the University Hospital Center of Liege, we identified 247 patients requiring high-flow oxygen support within 24 hours of admission, who formed our control group. Data matching was performed based on the calculation of the propensity score, i.e the probability for a subject to be assigned to a particular group of treatment, derived from a binary logistic regression model including the following covariates: age, day-0 SOFA score, worst PaO_2_/FiO_2_ ratio within 24h following ICU admission, and several biological values within 48 hours of ICU admission (lymphocyte count, ferritin, D-dimers and CRP). A sample of 24 control patients matching the 8 MSC patients was selected.

### Statistical Analyses

Quantitative parameters are summarized using median and interquartile range, whereas qualitative parameters are summarized using numbers (n) and frequencies (%). Comparisons of quantitative parameters between the 2 patient groups, MSC and control, were performed using the nonparametric Kruskal-Wallis test in the whole cohort and the non-parametric Wilcoxon signed ranks test in the matched sample. Qualitative parameters were compared between the two groups using the Fisher test in the whole cohort and the Mc Nemar test in the matched sample. The results were considered significant at the uncertainty level of α = 5% (p < 0.05).

Calculations were done using SAS (version 9.4, SAS institute, Cary, NC) and RStudio (version 3.6.2, Foundation for Statistical Computing, Vienna, Austria).

## Results

### Patients and Treatment

Between June 2020 and September 2021, 8 patients admitted to the ICU were enrolled to receive MSCs (7 men and 1 woman, aged 41 to 68 years). Their demographic characteristics are reported in **Table 1**. All patients had severe COVID-19 ARDS characterized by a WHO ordinal scale of severity score for COVID-19 of 6 (7/8) and 8 (1/8) (29). The median PaO_2_/FiO_2_ ratio was measured at 85.5 (IQR 77.9-93.4). At inclusion, seven patients were receiving HFNO and 1 patient required mechanical ventilation. Median SOFA score was 4 [IQR 3-5] and biological markers of inflammation were high (median CRP 171.7 mg/L [IQR 127.5-212.2], median ferritin 1,434.4 µg/L [IQR 756.4-2,742.4]), as were D-dimer levels (median 679.0 [IQR 501.5-1,713.0]). The time from first detection of SARS-CoV-2 (nasopharyngeal PCR) to admission to the hospital was 0-14 days. All included patients received their first dose of MSCs within 2 days of ICU admission.

**Table 1:** Patient baseline characteristics.

	Normal values	MSC group (n=8)	Matched control group	n	*p* ^1^	Whole control cohort	n	*p^2^*
Age (years)	–	50 (43-58)	54 (49.5-63)	24	0.089	67 (58-73)	247	0.0016
BMI (kg/m²)	–	31.5 (26.5-35.4)	30.1 (24.1-32.8)	19	0.34	28.7 (25. 7-31.8)	227	0.25
PaO2/FiO2	–	85.5 (77.9- 93.4)	86.5 (60.6-102.9)	24	0.89	76.6 (64.0-102.9)	242	0.33
Lymphocyte count (x10^9^/L)	1.10-3.70	0.82 (0.59-0.88)	0.77 (0.49-1.06)	24	0.90	0.63 (0.41-0.90)	247	0.34
SOFA score	–	4 (3-5)	4 (3-5)	24	0.81	4 (3-6)	182	0.54
CRP (mg/L)	< 5.0	171.6 (127.5- 212.2)	126.5 (62.2-216.5)	24	0.54	119.4 (63.3- 197.1)	247	0.31
Ferritin (µg/L)	22-275	1434.4 (756.4-2742.4)	1962.6 (990.1-2875.1)	24	0.78	1523.5 (740.5- 2677.7)	132	0.97
D-dimers (µg/L)	< 500	679.0 (501.5-1713.0)	1641.5 (628.5-1641.5)	24	0.098	1047.5 (599.0- 2043.0)	206	0.38
Creatinine (mg/dL)	0.55-1.18	0.83 (0.47- 1.15)	0.84 (0.72-1.03)	24	0.39	0.85 (0.67- 1.15)	247	0.37
Viral load (cycle threshold)	–	28.63 (24.05-29.90)	28.29 (20.68-32.11)	12	0.90	25.34 (20.83-29.09)	139	0.26

Values are expressed as median (P25-P75). p^1^ refers to comparisons between the MSC and matched control groups, whereas p^2^ refers to comparisons between the MSC group and the whole control cohort. BMI, body mass index; SOFA, Sepsis-related Organ Failure Assessment; CRP, C-Reactive Protein.

All patients received dexamethasone (6 mg/day during 10 days) and intermediate dose anticoagulation (enoxaparin 0.5 mg/kg/12h, unless a therapeutic dose was otherwise indicated). Seven patients received the 3 scheduled MSC doses; 1 patient did not receive the third dose after the demonstration of a cerebrovascular ischemic lesion. The median MSC dose (before thawing) was 3.15 x10^6^/kg per dose (range 2.41-4.47 x10^6^/kg), so that the post-thaw MSC dose of 1.5-3 x10^6^ viable cells/kg was reached on each occasion. Post-thawing viability ranged from 56 to 93% (median 76%). Mixed lymphocyte reaction (MLR) assays confirmed the immunosuppressive potency of MSCs post-thawing, with inhibition of lymphocyte proliferation (compared to control without MSCs) ranging from 32 to 70% (median 49%).

We compared these patients with the 247 patients admitted to the ICU of our institution during the same period who required HFNO within the first 24 hours of admission. The only difference in their baseline characteristics was age, with patients in the MSC group being younger (median 50 yrs [IQR 43-58] vs 67 yrs [IQR 58-73] in the control cohort; p=0.0016). The other baseline characteristics (day-0 SOFA score, worst PaO_2_/FiO_2_ ratio within 24h following ICU admission, lymphocyte count, ferritin, D-dimers, CRP, creatinine, viral load and body mass index) were similar between the two groups. As expected, no significant difference was observed between the MSC group and the control group of 24 patients selected after matching (**Table 1**).

### Outcomes

#### Safety

MSC infusions were well tolerated. We did not observe any significant change in parameters at the time of MSC infusion and immediately afterwards, nor any sign of allergy. One patient was diagnosed with multifocal ischemic cerebral lesions after the second MSC dose and did not receive the third dose. Further evaluation revealed a splenic infarction and an aortic endocarditis occurring on a bicuspid valve, that was considered the embolic source responsible for the stroke. This event was deemed not related to the MSC infusions. No adverse event related to MSCs was reported.

#### Efficacy

**Figure 1:**
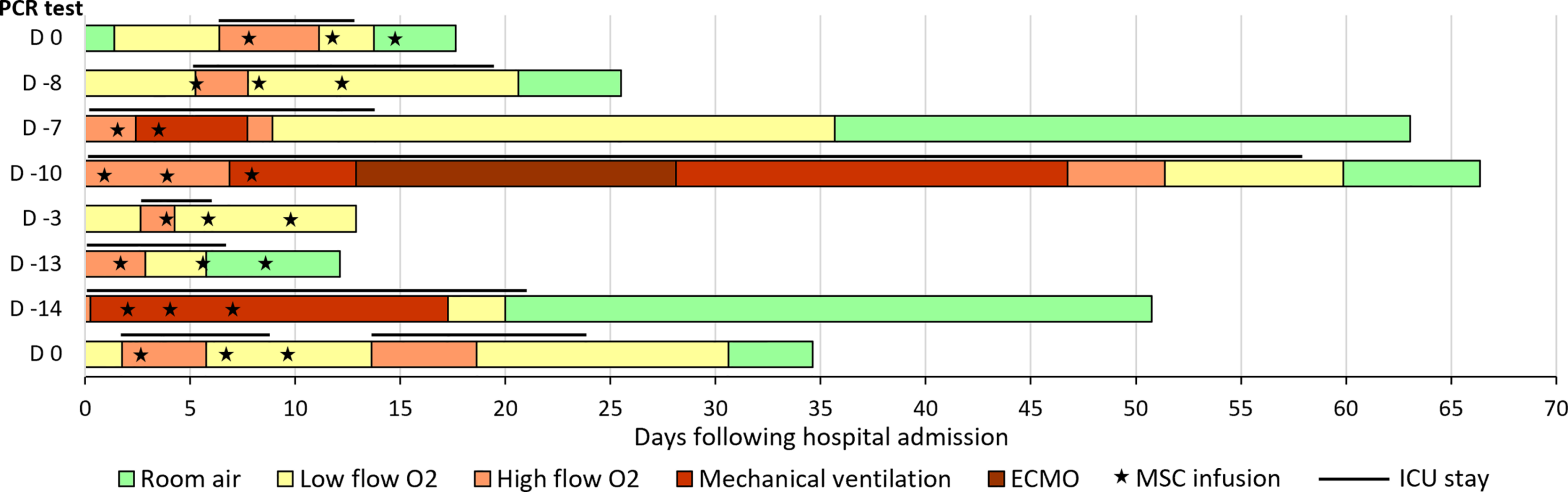
Evolution of oxygen support in patients treated with BM-MSCs. The bars represent the length of hospitalization (in days), and the left column indicates the day of the first positive PCR test, with day 0 being the day of hospital admission. The lines above the bars represent the length of ICU stay, and the “star” symbols represent MSC administrations. ECMO, extracorporeal membrane oxygenation; MSC, mesenchymal stromal cells; ICU, intensive care unit.

**Figure 2:**
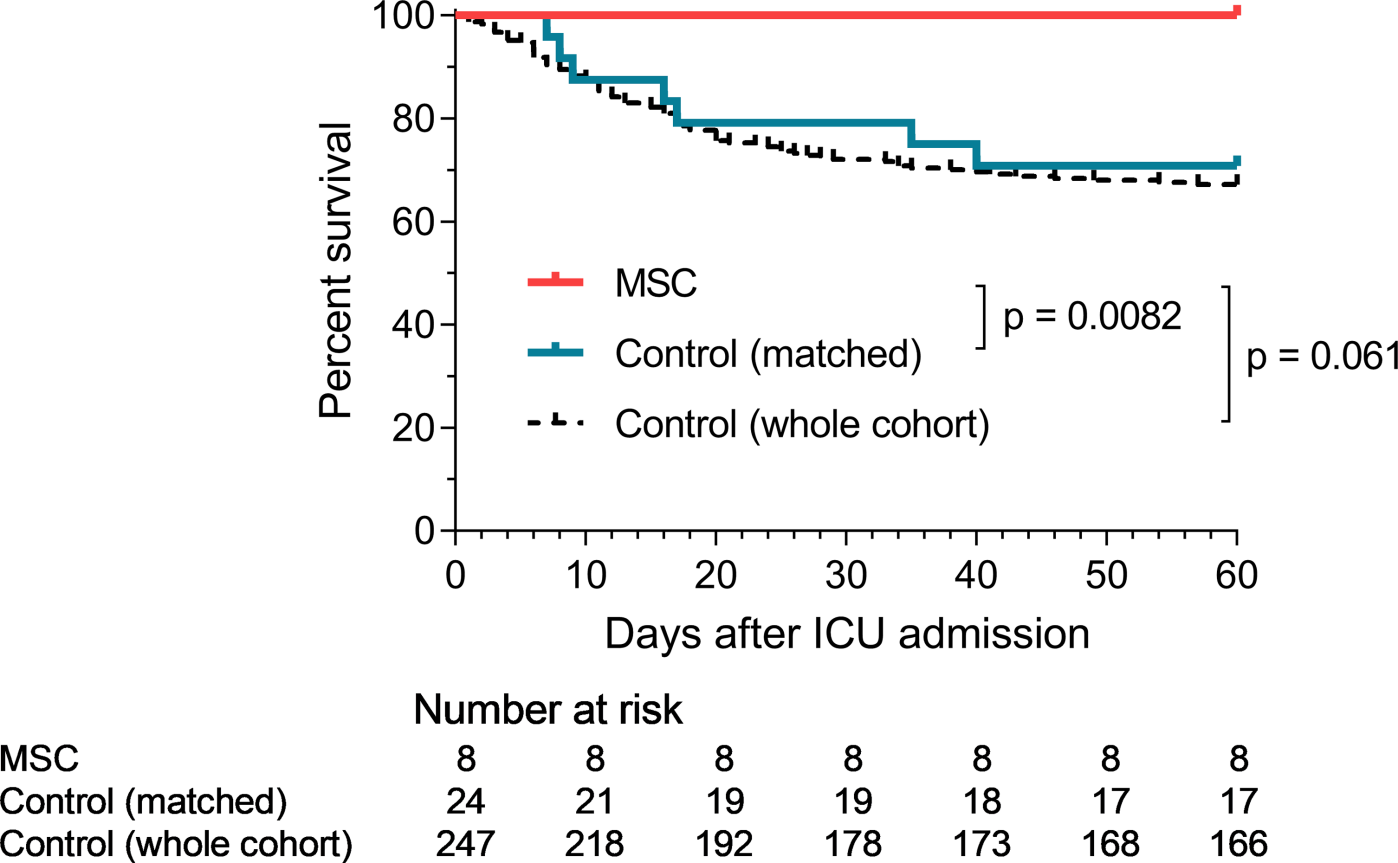
Survival curves in MSC and control groups. p-values refers to comparisons between the MSC and control groups at day 60 (using the Fisher test for non-matched analysis and Wilcoxon test for the matched analysis).

Most patients experienced rapid improvement in respiratory exchange after MSC infusions. Of the 7 patients requiring HFNO at inclusion, 2 required mechanical ventilation after MSC infusions, including one who required ECMO. Ultimately, all patients treated with MSCs had a favorable outcome, and none died (**Figures 1**, **2**). Median durations of hospital stay and ICU stay were 30 days (range 13-109 days) and 12 days (range 3-58 days), respectively. Of note, one patient, who initially showed a favorable course after MSC injections, had a secondary deterioration in respiratory parameters due to a pneumothorax, and required readmission to the ICU (thus prolonging the length of hospital and ICU stay).

We retrospectively compared the outcomes of these MSC-treated patients with those of our cohort of 247 unmatched control patients (**Table 2**). No significant difference was observed between the survival of the MSC group and the unmatched whole control cohort (**Figure 2**).

**Table 2:** Clinical and biological outcomes.

	MSC group (n=8)	Matched control group (n=24)	*p* ^1^	Whole control cohort (n=247)	*p^2^*
28-d mortality	0 (0%)	5 (20.8%)	0.025	65 (26.3%)	0.21
60-d mortality	0 (0%)	7 (29.2%)	0.0082	79 (32.0%)	0.061
ICU-free days	14 (7-22)	19 (0-24)	0.92	13 (0-23)	0.64
Need for MV	3 (37.5%)	11 (45.8%)	0.56	109 (44.1%)	1.00
Invasive ventilation-free days	11 (0-23)	0 (0-25)	0.50	0 (0-17)	0.54
HFNO support-free days	22 (11-27)	19 (0-25)	0.12	16 (0-24)	0.13
O2 support-free days	19 (9-25)	18 (0-25)	0.38	15 (0-24)	0.35
Need for noradrenaline	2 (25.0%)	11 (45.8%)	0.13	97 (39.3%)	0.49
Day-7 CRP (mg/L)	33.2 (6.3-59.8)	20.8 (10.6-92.1)	0.25	35.8 (12.2-94.5)	0.36
Day-7 D-dimers (µg/L)	821.0 (362.0-1,305.0)	3,553.0 (1,155.0-6,433.5)	0.0085	1,315.0 (675.0-3,425.0)	0.14

Values are expressed as median (P25-P75) for quantitative parameters and number (frequencies) for qualitative parameters. p^1^ refers to comparisons between the MSC and matched control groups, whereas p^2^ refers to comparisons between the MSC group and the whole control cohort. ICU, intensive care unit; MV, mechanical ventilation; HFNO, high flow nasal oxygen.

We then performed the same analyzes after matching (8 MSC patients vs 24 control patients). When comparing these matched patients, day-28 and day-60 survival was significantly superior in the MSC group (100% vs 79.2%, p = 0.025, and 100% vs 70.8%, p = 0.0082, respectively) (**Table 2** and **Figure 2**). No significant difference was observed in the percentage of patients requiring mechanical ventilation (37.5% vs 45.8%, p = 0.56), nor in the number of day-28 invasive ventilation-free days (median 11d [IQR 0-23] in the MSC group vs 0d [IQR 0-25] in the control group, p = 0.50), day-28 HFNO-free days (median 22d [IQR 10.5-26.5] in the MSC group vs 19d [IQR 0-25] in the control group, p = 0.12) or day-28 oxygen support-free days (median 19d [IQR 8.5-24.5] in the MSC group vs 18d [IQR 0-24.5] in the control group, p = 0.38). Day-28 ICU-free days was also similar in both groups (median 14d [IQR 6.5-22.0] in the MSC group vs 19d [IQR 0-23.5] in the control group, p = 0.92) (**Table 2**).

**Figure 3:**
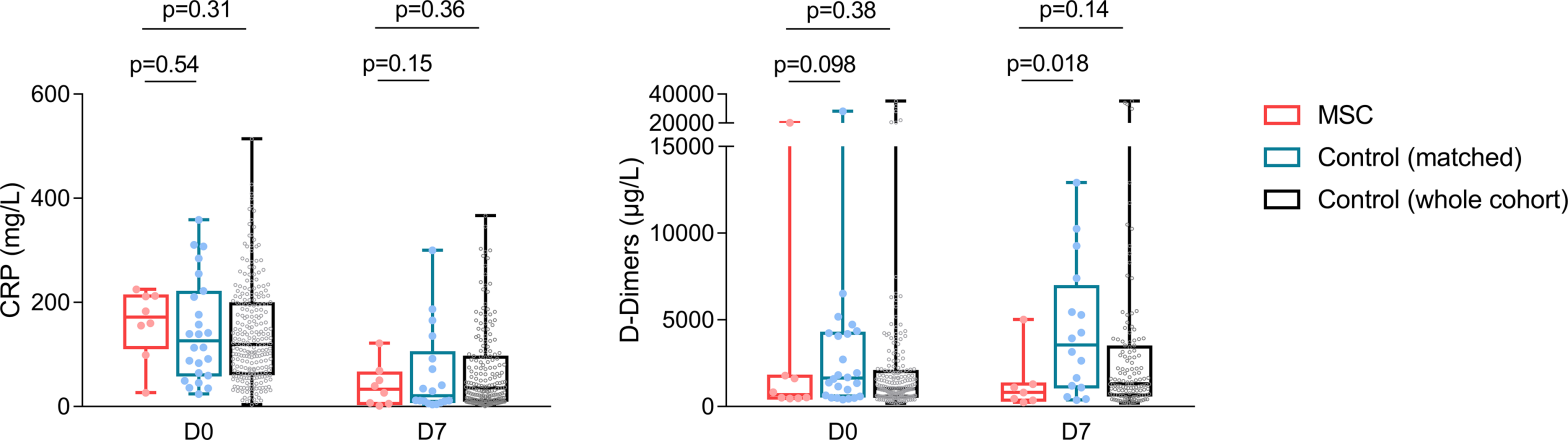
Day-0 and day-7 CRP and D-dimer values in the MSC and control groups. Boxes extend from the 25th to 75th percentiles, and the line in the box is plotted at the median, while whiskers represent minimum and maximum, and points represent individual values.

When analyzing day-7 biological inflammatory parameters, significantly lower D-dimer values were observed in the MSC group (median 821.0 µg/L [IQR 362.0-1305.0] vs 3,553 µg/L [IQR 1,155.0-6,433.5] in the control group, p = 0.0085), while CRP values remained similar between the 2 groups (median 33.2 mg/L [IQR 6.3-59.8] in the MSC group vs 20.8 mg/L [IQR 10.6-92.1] in the control group, p = 0.25) (**Table 2**, **Figure 3**).

## Discussion

The COVID pandemic has resulted in significant morbidity and mortality worldwide, and is a significant burden on health care, particularly ICUs. Although significant regional, temporal and center-dependent discrepancies in outcomes have been reported, mortality of critically ill patients in ICU remains high (1). The overall case fatality rate for mechanically ventilated patients is 40-45%, with even worse results in pooled analyses of elderly patients or those with comorbidities (30). In addition, among patients surviving after severe COVID-19, more than 50% experience a substantial reduction in activities of daily living and physical performance (31, 32). The societal cost of ARDS was estimated in a recent systematic review to be approximately €70,000 per case (33). It is therefore essential to identify new therapies for the prevention and treatment of severe forms of COVID-19.

Given their anti-inflammatory and regenerative properties, MSCs represent a promising therapy for many immune and inflammatory disorders, and particularly pulmonary diseases. Indeed, several studies have demonstrated the early trapping of MSCs in the lung microvasculature, where they interact with cytolytic cells and phagocytes and secrete soluble factors responsible for paracrine and systemic effects (34, 35). MSC therapy has demonstrated its efficacy in several animal models of ARDS, induced mostly by LPS, bacteria, bleomycin or hyperoxia, but also by influenza A H5N1 or after ventilator-induced lung injury (36). In these models, MSC administration mitigated inflammation, resulting in reduced pulmonary edema, increased alveolar fluid clearance, decreased epithelial and vascular permeability, improved oxygenation and longer survival. These improvements were associated with decreased levels of several inflammatory mediators (IL-1β, IL-6, IL-8, IFN-γ and TNF-α) and increased levels of anti-inflammatory and trophic factors (IL-1RA, IL-10, prostaglandin E2, TSG-6). MSCs also induced a shift from pro-inflammatory M1 to anti-inflammatory M2 monocytes/macrophages with enhanced phagocytic capacities and from pro-inflammatory Th17 cells to Tregs. Prior to the COVID-19 pandemic, 3 small phase 1/2 clinical trials had evaluated the use of MSCs in ARDS, but, despite encouraging preliminary results, neither adipose tissue-derived (AT)-MSC (1x10^6^/kg) nor BM-MSCs (10x10^6^/kg) have so far demonstrated their ability to significantly improve survival in ARDS (37–40).

Very early in the COVID-19 pandemic, several groups conducted small phase 1 trials evaluating the use of MSCs, mostly derived from umbilical cord (UC), in patients with COVID-19 of varying severity, confirming the absence of adverse effects and suggesting favorable outcomes (25, 41–43). A few groups described outcomes in series of critically ill COVID-19 patients in intensive care, in whom MSC therapy was also found to be safe (44–47). Five randomized trials evaluating UC-MSCs in COVID-19 patients have been reported since then, with heterogenous methodology and results. Lanzoni et al. (48) and Monsel et al. (49) included only patients with ARDS, requiring at least high-flow oxygen, whereas the patient groups were more disparate (or less well described) in the 3 other studies (50–52). MSC dose and administration scheme also differed between the studies, with overall lower doses than in our trial. Outcomes were favorable in three of these trials, with improved survival in two trials (48, 51); Shi et al. however only reported radiological and functional outcomes (50). On the contrary, Monsel et al. found that 3 infusions of 1x10^6^ UC-MSCs/kg in the early phase of COVID-19 ARDS did not improve PaO2/FiO2-ratio between D0 and D7, which was the primary endpoint. No significant differences were observed regarding the secondary endpoints either, including day-28 ventilation-free days (median 17 vs 12 days) nor day-28 mortality (26.3% vs 18.2%) (49).

In our study, 3 infusions of 1.5-3x10^6^ BM-MSC/kg in the early phase of COVID-19 ARDS were safe, and resulted in 100% survival, which was significantly superior to that of matched control patients treated in our institution during the same period. However, MSC therapy did not significantly reduce the ICU-free days, need for hemodynamic or invasive respiratory support, and duration of oxygen support. We observed significantly lower day-7 D-dimer values compared to those in the matched control patients, which is an important finding since high levels of D-dimers are associated with worse outcomes in COVID-19 ARDS (11, 53).

Unlike most studies that used UC-MSCs in COVID-19 ARDS, we chose to use BM-MSCs. Indeed, few safety data on MSCs in COVID-19 were available when we initiated this study, and BM-MSCs have been more widely used in clinical studies (notably in GVHD, Crohn’s disease or organ transplantation), with an excellent safety profile. In addition, the procoagulant nature of MSCs, through tissue factor (TF) expression, was a concern given the known tendency for thrombosis in COVID-19, and BM-MSCs, which have low TF expression, activate coagulation to a much lesser extent than UC- and especially AT-MSCs (54–56). Several studies have now reported no increased thrombotic risk associated with UC-MSCs in COVID-19, which may be related to the wide use of higher doses of low molecular weight heparin in these patients (57). Similarly, patients in our study received at least intermediate-dose prophylactic anticoagulation as part of our institutional protocol in COVID-19 patients admitted to the ICU. Importantly, we observed lower D-dimer concentrations at D7 in the MSC group compared with our matched control group, confirming the absence of a systemic or prolonged procoagulant effect of BM-MSCs in this setting. The hemocompatibility of MSCs is crucial for safety in case of intravenous use, especially in hypercoagulable situations, and further evaluation of TF expression and hemocompatibility might be required to assess the suitability of MSC products (especially when isolated from other sources that the BM) in future clinical studies (56, 58). Furthermore, it has been shown that MSC infusion could be accompanied by a transient elevation of IL-6, and that this effect was more pronounced with UC-MSCs than BM-MSCs (54, 59). Given the importance of IL-6 in the pathophysiology of COVID-19-related ARDS, this could have a negative impact on patient outcome. MSCs from different origins also have different immunomodulatory effects, and this could influence the efficacy of MSC therapy in COVID-19, but further studies are required to assess the importance of this parameter. Nevertheless, the procoagulant effects and IL6 secretion of MSCs may limit the benefits of their immunomodulatory and regenerative properties, and BM-MSCs may be a better choice than UC-MSCs in these regards.

The optimal dose and schedule of administration of MSCs is unknown. We chose to repeat MSC infusions 3 times at 3-day intervals given the short persistence of these cells after systemic administration, and infused higher doses of MSCs (1.5-3x10^6^/kg/injection) than in most other trials, based on preclinical and preliminary clinical results in ARDS suggesting that higher doses (total dose up to 10x10^6^/kg) might provide increased clinical benefits (38). However, we lack dose-escalation studies to assess whether this approach is optimal. Another potentially important parameter is the impact of cryopreservation and freezing-thawing on the viability and therapeutic properties of MSCs. Cryopreserved “off-the-shelf” MSCs are easy to use and immediately available, which is particularly important in acute situations such as rapidly progressive COVID-19 ARDS. However, immediately after thawing, MSCs exhibit partially reduced immunosuppressive properties *in vitro*, as well as reduced homing properties and increased complement-mediated lysis and coagulation activation (40, 60). Whether these alterations negatively impact the MSC therapeutic effect has yet to be evaluated in clinical trials, and probably depends on the MSC product and the mechanisms of action of MSCs in each disease. Indeed, MSC viability is essential for several of their functions, but phagocytosis of apoptotic MSCs by host immune cells has also been identified as an important mechanism of action (34). Suboptimal viability of MSCs after thawing has been suggested to be responsible for the failure of the START-1/2 trial in ARDS, as outcomes seemed more favorable in patients receiving BM-MSCs with intermediate (57-69%) or high (80-85%) viability (which is similar to the 56-93% viability in our study), compared to those receiving BM-MSCs with low viability (36-56%) (39). On the other hand, UC-MSCs did not improve COVID-19 ARDS in the study by Monsel et al. despite a high post-thawing viability (≥ 70%), suggesting that a high viability is important but not sufficient for the therapeutic efficacy of MSC in COVID-19 ARDS. Finally, we chose to treat patients within 48 hours of ICU admission, in the early phase of ARDS, in an attempt to stop the inflammatory process before it becomes irreversible. Contrary to the negative trial of Monsel et al. (49), most of our patients were receiving HFNO at inclusion (only one received invasive mechanical ventilation at the time of MSC infusion), indicating a less severe condition or an earlier intervention, which might account for the better outcomes.

The main limitations of this study are the small sample size and the lack of randomization. Inclusion in our study was hampered by the concurrent availability of other phase 2 or 3 studies in our hospital, and by the kinetics of arrival of COVID-19 cases in waves. Moreover, since the introduction of SARS-CoV-2 vaccination (with excellent coverage in Belgium), the number of severe cases has decreased, and it is now mainly immunocompromised patients who remain at risk of critical COVID-19 infection, but they were initially excluded from our study. For the continuation of the study, given the good safety of MSCs and the encouraging results in immunocompetent patients, we amended the study protocol to also include immunocompromised patients. The design of the study, which is non-randomised, is also an important limitation, and does not allow us to conclude that BM-MSCs are effective in this indication. However, the 100% survival rate in patients treated with MSCs is particularly high compared to the figures reported in the literature for patients with the same severity of disease and compared to our large control group treated concurrently in our institution. The main strengths of this study are: (1) the use of BM-MSCs, unlike previous studies that have predominantly used UC-MSCs (only one small study reported the benefit of pooled BM-MSCs in 5 critically-ill COVID patients), (2) the description of a relatively homogeneous group of patients in terms of respiratory parameters and adjuvant treatment (only dexamethasone in all patients) and (3) the comparison to a large database of similarly treated patients at the same institution.

In conclusion, we report positive results of BM-MSCs on survival of patients with severe COVID-19, along with satisfactory safety, which will need to be confirmed in randomized placebo-controlled trials. Several issues will need to be addressed in future studies, including the optimal treatment conditions (timing, dose, provenance of MSCs), the interaction between MSCs and concomitant treatments (dexamethasone, anti-IL6 or JAK inhibitors), and the exact mechanisms of action of MSCs in COVID-19 ARDS. Furthermore, it will be interesting to investigate whether efficacy is maintained in immunocompromised patients in the continuation of our trial. Indeed, it has been suggested that a key factor determining the efficacy of MSC therapy is the ability of host immune system to interact with MSCs (notably the ability of lung-resident cytotoxic and phagocytic cells to induce MSC apoptosis and to engulf apoptotic MSCs) (34). Ultimately, the scientific and medical efforts deployed to fight this COVID-19 pandemic may lead to advances in other pathologies, and it would be interesting to further investigate the interest of BM-MSCs in non-COVID ARDS and in pulmonary fibrosis.

## Data Availability Statement

The study protocol and individual participant data that underlie the results reported in this article, after deidentification, can be shared with investigators whose proposed use of the data has been approved by the ethic committee of the University Hospital of Liège. Data can be provided for meta-analysis or other projects comparing MSC efficacy in COVID-19. Requests should be addressed to Yves Beguin.

## Ethics Statement

The study protocol conformed to the ethical guidelines of the 1975 Declaration of Helsinki and was approved by the local Ethics Committee of the University Hospital of Liège, the centralized Ethics Committee of the Cliniques Universitaires Saint-Luc, and the Belgian Federal Agency for Medicines and Health Product (EudraCT: 2020-002102-58; ClinicalTrials.gov identifier: NCT04445454). Signed informed consent was obtained from participating subjects, or - if impossible (clinical condition precluding capacity to consent) - from his/her legal representative.

## Author Contributions

CG and YB designed the study. NL, BL, and BM contributed to conception and designing of the study. NL and BL recruited the patients. CL, AB, VB, and EB isolated and cultured the MSCs. CG, NL, BL, and BM analysed and interpreted the data. MT organized the database and extracted the data. ND performed the statistical analyses. CG wrote the first draft of the manuscript with contributions of NL. All authors contributed to manuscript revision, read and approved the submitted version.

## Funding

This study was supported by funds from Leon Fredericq Foundation.

## Conflict of Interest

The authors declare that the research was conducted in the absence of any commercial or financial relationships that could be construed as a potential conflict of interest.

## Publisher’s Note

All claims expressed in this article are solely those of the authors and do not necessarily represent those of their affiliated organizations, or those of the publisher, the editors and the reviewers. Any product that may be evaluated in this article, or claim that may be made by its manufacturer, is not guaranteed or endorsed by the publisher.
