# Supplementary material for: Bone Marrow-Derived Mesenchymal Stromal Cell Therapy in Severe COVID-19: Preliminary Results of a Phase I/II Clinical Trial
Source: Front Immunol. 2022 Jul 4;13:932360. doi: 10.3389/fimmu.2022.932360 (PMC9291273; doi:10.3389/fimmu.2022.932360)
Supplement: Supplementary file 1 [file DataSheet_1.docx]

Supplementary Material

# Supplementary Data

**Bone marrow collection and manufacturing process**

Bone marrow collection

Bone marrow (50 ± 10 ml) from eligible donors was harvested from the posterior iliac crest under local anesthesia in sterile conditions, placed in a sterile bag containing heparin, and labeled.

Processing of the bone marrow

Mononuclear cells were isolated (using a ficoll isolation procedure), washed twice with a solution of 97.5 % NaCl 0.9% and 2.5 % human albumin 20%, then suspended in a solution of 90% DMEM-LGGLX medium (Fisher-Bioblock, Invitrogen, Merelbeke, Belgium) and 10% γ-irradiated fetal bovine serum (FBS, Hyclone, Perbio Sciences, Smithfield, UT, USA). All these operations were performed with a fully automated and closed capability system. The cell suspension was then transferred to the ATMP production staff to be processed in a class A in B environment.

Initiation of in vitro cultures (P0) and replacement of the culture medium

The cell suspension was seeded in sterile tissue culture flasks (T-175 or CellSTACK from Corning) at a density of 160,000 cells/cm² under laminar air flow and then incubated at 37°C, 5% CO_2_ and 90 % relative humidity. Adherent precursors were selected by removing non-adherent cells three days after culture initiation. MSC were then expanded by replacement of the medium (90% DMEM-LGGLX medium and 10% γ-irradiated FBS) twice a week until passage 1. The medium was also replaced three days after passage 1, after passage 2 and at thawing.

Passages 1 and 2

On day 14, MSC were at about 70% confluence and were trypsinized and replated at a lower density (4,000 cells/cm^2^) to allow further cell expansion. Cells were first washed with phosphate-buffered saline (PBS), before incubation with TryplE (Life Technologies, Bleiswijk, The Netherlands) for five minutes at 37°C. Then, they were collected, washed and resuspended in culture medium. After dilution at the appropriate density in the complete culture medium (90% DMEM + 10% γ-irradiated FBS), cells were replated in Cellstacks and incubated at 37°C, 5% CO_2_ and 90 % relative humidity. On day 21, cells were again nearly confluent and subjected to a second passage, following the same modalities. At this step, batches could be divided in different subbatches.

Harvest and freezing

At day 28, cells were trypsinized (as described above), washed and resuspended (at the double concentration of 2x10^6^ cells/ml compared to the final frozen concentration of 1x10^6^ cells/ml) in harvesting solution (95% NaCl 0.9% and 5% human albumin 20%). Then, the cell suspension was mixed volume to volume with the freezing solution (60% NaCl 0.9%, 20% human albumin 20% and 20% DMSO 99.99%). The cell suspension was finally frozen according to a controlled preprogrammed temperature curve and stored in gaseous nitrogen.

**Phenotypic characterization of MCS**

Analysis of cell-surface molecules was performed on MSC cultures using flow cytometry. Harvested cells were washed with PBS containing 5% HSA. Around 2x10^5^ cells were resuspended in 90 µL PBS containing 5% HSA, and incubated for 10 min on ice in the dark, with the following MAb: CD45-FITC, human; clone REA 747 - CD73-APC, human, Clone REA804, - CD90-VioBlue, human, Clone REA897, - CD105-PE-Vio770, human, Clone REA794, - Anti-HLA-DR-APC, human, Clone REA805, - CD14-VioBlue, human, clone REA599 - CD34-PE-Vio770, human, Clone REA1164 - CD3-FITC, human, Clone REA613 - CD29-APC, human, Clone REA1060 - CD44-PE-Vio770, human, Clone REA690 - CD166-VioBright FITC human Clone REA442, - REA Control (S)-FITC, Clone REA293, - REA Control (S)-APC, Clone REA293, - REA Control (S)-VioBlue Clone REA293 - REA Control (S)-PE-Vio770, Clone REA293 - REA Control (S)-VioBright FITC, Clone REA293 (Miltenyi biotec). Data were acquired on a Macsquant Flow Cytometer (Miltenyi Biotec) by collecting a minimum of 10,000 events and analyzed with the Macsquantify software.

**MSC immunosuppression assays**

One x10^4^ MSCs were plated in triplicates in round-bottom 96-well plates (Becton-Dickinson) in a total volume of 100 μl of RPMI 1640 medium supplemented with 10% FBS,100 U/ml penicillin, 100 mg/ml streptomycin, L-glutamine (2 mM) (all from Lonza), sodium pyruvate (100 mM), non-essential amino acids (NEAA) (100 mM) and 5×10^-5^ M β-mercaptoethanol (β-ME) (all from Gibco, Merelbeek, Belgium). After 4-hour incubation, MSC were irradiated at 25 Gy using a ^137^Cs source (GammaCell 40, Nordion, Ontario, Canada).

Allogeneic human peripheral blood mononuclear cells (PBMCs) were isolated from a blood sample (healthy volunteer donor) by Ficoll Paque Plus^®^ density gradient. PBMCs (5x10^4^ or 1x10^5^) were then added to wells in a total volume of 200 μl containing or not irradiated MSCs, in the presence of anti-αCD3/CD28 microbeads (Invitrogen, Dynal A/S, Oslo, Norway). Co-cultures without anti-αCD3/CD28 microbeads were used as controls. Cells were then incubated at 37°C in 5% humidified air for 4 days. Cell cycle analysis of PBMC stimulated or not with anti-αCD3/CD28 microbeads and cultured during 4 days with or without MSCs were performed using the CycleTEST Plus^®^ DNA Reagent Kit (Becton Dickinson). The percentage of cells in the different phases of the cell cycle was determined with the Macsquant Software (Miltenyi) or the Modfit Software (Becton Dickinson). The effect of MSCs on PBMC stimulation responses was calculated as percentage suppression compared with the proliferative response in the positive control without MSC (+- standard deviation of the mean). The positive control was set to 0% suppression.

**Population doubling level (PDL) calculation**

PDL was calculated according to the formula PDL= 3.322 (log Y – log I) where Y = number of cells harvested and I = number of cells inoculated at P1.

# Supplementary Figures and Tables

**Supplementary Table 1.** Quality controls and release criteria of MSCs.

| **Test** | **Method** | **Release criteria** |
| --- | --- | --- |
| **Drug Substance** | | |
| Morphology | Microscopic observation | Fibroblastic |
| Identity | Phenotype by FACS | CD90>95%  CD105>95%  CD73>95%  CD29>90%  CD44>90%  CD166>90% |
| Purity | Phenotype by FACS | CD14<2%  CD34<2%  CD45<2%  CD3<1% |
|  |  | CD14 + CD34 + CD45 + CD3 < 2% |
| Karyotype | Cell culture | Absence of chromosomal structure and / or number abnormalities |
| Viability | Nucleocounter NC-200 | ≥80% |
| Immunosuppressive properties (potency test) | MLR by FACS | > 25% inhibition of the proliferation of activated PBMCs |
| Cell aggregates | Nucleocounter NC-200 | < 25 % |
| **Drug Product** | | |
| Visual inspection | European Pharmacopoeia 2.9.20 | Absence of visible particle |
| Sterility test | European Pharmacopoeia 2.6.27 | Culture negative at the limit of detection |
| Mycoplasma test | European Pharmacopoeia 2.6.7 | Absence of mycoplasma |
| Endotoxin test | European Pharmacopoeia 2.6.14 | < 2.5 UI/ml |
| Freezing temperature curve | Control of the freezing temperature curve | Conform to the programmed temperature curve |

FACS: Fluorescence activated cell sorting; MLR: Mixed Lymphocyte Reaction.
